# RBT‐1, a “preconditioning” agent, mitigates syndecan‐1 shedding in patients undergoing “on pump” cardiac surgery and following experimental AKI

**DOI:** 10.14814/phy2.70218

**Published:** 2025-02-04

**Authors:** Ali C. M. Johnson, Richard A. Zager

**Affiliations:** ^1^ Renibus Therapeutics Southlake Texas USA; ^2^ Fred Hutch Cancer Center Seattle WA USA; ^3^ The University of Washington Seattle WA USA

**Keywords:** acute kidney injury, endotoxemia, glycocalyx, isoproterenol, Nrf2, preconditioning, syndecan‐1

## Abstract

During systemic stress, syndecan‐1 (SDC‐1) shedding into plasma results, implying endothelial damage. RBT‐1, a “preconditioning” agent, has been shown to mitigate postoperative complications following cardiac surgeries. This study assessed whether RBT‐1 preconditioning attenuated SDC‐1 shedding in these patients, implying a vascular protective effect. Patients (*n*, 112) were randomized to receive low‐dose RBT‐1, high‐dose RBT‐1, or placebo 24–48 h prior to surgery. Plasma samples were obtained before and 2 days postsurgery and assayed for SDC‐1 (ELISA). To gain further insights, male CD‐1 mice were subjected to acute renal injuries, and RBT‐1's impact on plasma SDC‐1 increases, vascular/aortic stress responses (NGAL/KIM‐1/IL‐6 gene induction), and two vascular cytoprotective pathways (Nrf2; ferritin) were assessed. Baseline plasma SDC‐1 levels did not differ between patient groups. The placebo group developed an approximate 50% plasma SDC‐1 (ng/mL) increase (*p*, 0.012). Conversely, no significant SDC‐1 increases were seen in the RBT‐1 treatment groups. Experimental injury markedly increased plasma SDC‐1 concentrations, and these were significantly blunted by RBT‐1 preconditioning. RBT‐1 also mitigated AKI‐induced aortic NGAL/KIM‐1/IL‐6 mRNA increases, activated aortic Nrf2, and increased vascular ferritin levels. RBT‐1 preconditioning diminishes SDC‐1 release and upregulates vascular ferritin and Nrf2. Hence, RBT‐1 preconditioning can confer select vasoprotective effects.

## 1 INTRODUCTION

Transient bouts of tissue injury, for example, as induced by ischemia, can trigger adaptive responses that can protect tissues against subsequent insults. This phenomenon, commonly referred to as “ischemic preconditioning”, was first demonstrated in kidney (Zager et al., 1984), and subsequently it has been documented in virtually every organ (e.g., (de Paula et al., 2022; Koti et al., 2003; Lee et al., 2018)). These observations have stimulated efforts to identify safe methods to mimic “ischemic preconditioning” in humans. If a safe and effective method were identified, it could be used to protect patients who are about to undergo major cardiovascular surgeries or other high risk medical interventions.

One approach towards achieving this goal has been “remote preconditioning” (RP) in which repetitive bouts of limb ischemia, induced by inflating blood pressure cuffs, are performed shortly before major cardiovascular procedures (Anttila et al., 2016). However, inconclusive organ protection has been observed (Hausenloy et al., 2007, 2015; Meybohm et al., 2015; Pierce et al., 2017; Zarbock et al., 2015). A major factor that potentially limits efficacy is the short time window between RP and the start of surgery. Hence, there is virtually no time prior to surgery to permit the production of cytoprotective anti‐inflammatory and antioxidant proteins in target tissues.

We have sought an alternative strategy to safely induce a potent preconditioning response. To this end, RBT‐1, a combination product composed of proprietary stannic protoporfin (SnPP) and iron sucrose (FeS) has been studied (Johnson et al., 2017; Johnson & Zager, 2018; Zager, 2015, 2022; Zager et al., 2016; Zager & Johnson, 2021). RBT‐1 induces a transient pro‐oxidant signal which frees Nrf2 from its inhibitory binding protein, KEAP‐1 (e.g., ref. (Johnson et al., 2017)). This permits Nrf2 translocation from the cytosol to the nucleus where it activates gene transcription (Johnson et al., 2017; Johnson & Zager, 2018; Zager, 2022). Within 12–24 h of RBT‐1 infusion, a host of Nrf2‐dependent antioxidant and anti‐inflammatory proteins are upregulated in multiple organs (kidney, liver, heart, and lung; ref. (Johnson et al., 2017; Johnson & Zager, 2018; Zager, 2015, 2022; Zager et al., 2016; Zager & Johnson, 2021); unpublished data). In addition, the FeS component of RBT‐1 markedly increases tissue heavy chain (antioxidant) ferritin translation. As a result of these combined actions, synergistic protection against multiple experimental models of acute tissue damage has been observed (e.g., renal ischemia, nephrotoxic injuries, and hepatic ischemia (Johnson et al., 2017; Johnson & Zager, 2018; Zager, 2015, 2022; Zager et al., 2016; Zager & Johnson, 2021); oleate‐induced acute lung damage (unpublished data)).

An unresolved question is whether the intravenous infusion of RBT‐1 might also upregulate cytoprotective pathways directly within the vasculature, and if so, would vascular protection result? The endothelial glycocalyx, and its constituent protein, syndecan‐1 (SDC‐1), line the luminal side of blood vessels and modulate several key vascular functions, including the control of vascular permeability, inflammation, thrombosis, and cytokine signaling (Alphonsus & Rodseth, 2014; Liu et al., 2020; Pillinger & Kam, 2017; Svennevig et al., 2008). During critical illnesses, trauma, and major surgeries (e.g., “on pump” open heart procedures), endothelial SDC‐1 shedding into plasma results (Bruegger et al., 2009, 2011; de Melo Bezerra Cavalcante et al., 2016; Dixon et al., 2024; Gonzalez Rodriguez et al., 2017; Miyazaki et al., 2024; Passov et al., 2021; Pesonen et al., 2019; Puskarich et al., 2016; Salmito et al., 2024; Xu et al., 2021). Hence, plasma SDC‐1 has become a widely accepted biomarker of endothelial glycocalyx damage, as well as a predictor of subsequent adverse clinical outcomes (Bruegger et al., 2009, 2011; de Melo Bezerra Cavalcante et al., 2016; Dixon et al., 2024; Miyazaki et al., 2024; Pesonen et al., 2019; Salmito et al., 2024; Svennevig et al., 2008; Xu et al., 2021).

Given these considerations, we questioned whether RBT‐1 preconditioning might mitigate glycocalyx injury, and thus, blunt plasma SDC‐1 increases following “on pump” cardiac surgeries. To address this question, we measured plasma SDC‐1 levels in patients who participated in a recent double‐blind, placebo‐controlled phase 2 study which was conducted in subjects who underwent “on pump” cardiac surgery with and without RBT‐1 preconditioning (Lamy et al., 2024). In complementary mouse studies, we evaluated whether RBT‐1 preconditioning might mitigate SDC‐1 release following endotoxemia or nephrotoxic tissue damage. We also assessed whether RBT‐1 would activate Nrf2 and upregulate ferritin expression directly within vascular (aortic) tissues, potentially conferring cytoprotective effects. These combined clinical and experimental studies form the basis of this report.

## 2 METHODS

### 2.1 Plasma SDC‐1 levels before and after cardiac surgery: Impact of RBT‐1 preconditioning

A recent IRB approved multicenter, randomized, double‐blind, placebo‐controlled phase 2 clinical trial (NCT04564833; START) evaluated whether RBT‐1 preconditioning could decrease complications following “on pump” cardiac surgery (Lamy et al., 2024). In that study, 121 patients were randomized (1:1:1) to receive either placebo, low dose RBT‐1 (45 mg SnPP/240 mg FeS), or high dose RBT‐1 (90 mg SnPP/240 mg FeS) 24–48 h prior to surgery. There were no evidenced baseline demographic differences, including degrees of renal function, between the three groups. The results of that study implied significant clinical benefits, based on a post hoc hierarchical analysis of multiple clinical endpoints (Finkelstein & Schoenfeld, 1999; Pocock et al., 2012).

To assess whether this previously noted protection was associated with diminished SDC‐1 release, in the current study, paired plasma samples that had been collected before surgery (baseline) and 2 days after surgery were assayed for SDC‐1 by ELISA (ab46506; Abcam, Waltham, MA). Absolute SDC‐1 concentration (ng/mL) changes from pre‐ and post‐surgery were assessed. Paired samples were available for assay from 112 of the original 121 studied subjects (39, 36, and 37 subjects in the low dose RBT‐1, high dose RBT‐1, and placebo groups, respectively). Baseline demographics for the three patient groups that were assayed for SDC‐1 are presented in Table 1.

**TABLE 1:** Demographics and baseline characteristics for the patients evaluated for plasma syndecan 1 concentrations pre‐ and post‐surgery.

	High‐dose (*n* = 39)	Low‐dose (*n* = 36)	Placebo (*n* = 37)
Age, mean ± 1 SD, years	67.7 ± 10.8	63.9 ± 8.1	66.4 ± 8.2
Sex			
Female	8 (21%)	9 (25%)	10 (26%)
Male	30 (79%)	27 (75%)	29 (74%)
Race			
White	34 (89%)	30 (83%)	37 (95%)
Black	1 (3%)	3 (8%)	1 (3%)
Asian	2 (5%)	1 (3%)	1 (3%)
American Indian	1 (3%)	0 (0%)	0 (0%)
Other	0 (0%)	2 (6%)	0 (0%)
‐Country			
United States of America	27 (71%)	27 (75%)	27 (69%)
Canada	7 (18%)	6 (17%)	10 (26%)
Australia	4 (11%)	3 (8%)	2 (5%)
Weight, mean ± SD, kg	90.4 ± 18.0	99.5 ± 20.2	89.4 ± 18.7
Body mass index, mean ± SD, kg/m^2^	30.0 ± 6.5	33.3 ± 6.0	29.8 ± 5.2
EuroSCORE II, median (IQR)	1.7 (1.1–2.5)	1.1 (0.8–2.1)	1.5 (0.9–2.3)
Low risk (<3)	30 (79%)	30 (83%)	33 (85%)
Medium risk (3–6)	6 (16%)	3 (8%)	4 (10%)
High risk (≥6)	2 (5%)	3 (8%)	2 (5%)
Injury risk factors			
Age ≥ 65 years	25 (66%)	20 (56%)	22 (56%)
Diabetes mellitus requiring insulin	8 (21%)	5 (14%)	3 (8%)
Congestive heart failure	6 (16%)	4 (11%)	6 (15%)
Heart failure (NYHA III/IV) within 1 year prior to surgery	4 (11%)	2 (6%)	2 (5%)
Previous cardiac surgery with sternotomy	1 (3%)	1 (3%)	0 (0%)
Left ventricular ejection fraction ≤35%	4 (11%)	3 (8%)	2 (5%)
Estimated glomerular filtration rate ≥ 20 < 60 mL/min/1.73m^2^	11 (29%)	11 (31%)	6 (15%)
Preoperative anemia (hemoglobin <10 g/dL)	0 (0%)	0 (0%)	1 (3%)
Hospitalized for management of cardiac or pulmonary disease	10 (26%)	2 (6%)	7 (18%)
Surgery type			
CABG alone	23 (61%)	18 (50%)	18 (46%)
Valve alone	7 (18%)	12 (33%)	7 (18%)
CABG + Valve	8 (21%)	6 (17%)	14 (36%)

*Note*: Data are given as mean ± 1 SD or as percentages, rounded to the nearest whole number.

Abbreviations: CABG, coronary artery bypass graft; EuroSCORE, European system for cardiac‐operative risk evaluation; NYHA, New York Heart Association.

### 2.2 Ethics

Institutional review board approval was obtained centrally (Advarra; Columbia, MO USA) or locally based on center requirements (Junqueira et al., 2023; Lamy et al., 2024). Informed consent was obtained from each patient before enrollment. The protocol was approved by the relevant health authorities and institutional review boards at each site (date: November 6, 2020; project number: Pro00047629). Performance sites are listed in Acknowledgments sections. The trial was registered on ClinicalTrials. gov (NCT04564833) prior to study enrollment. It followed the Consolidated Standards of Reporting Trials (CONSORT) Harms 2022 and adaptive designs CONSORT extension reporting guidelines (Puskarich et al., 2016; Salmito et al., 2024). Standard procedures were followed for handling and processing records as per Good Clinical Practice (GCP) and the data management standard operating procedures of the contract research organization. Supplementary material related to this article and approvals can be found at https://doi.org/10.1016/j.eclinm.2023.102364. The institutional sites at which surgeries were performed are listed in this report's Acknowledgments section.

### 2.3 Mouse studies

Male CD‐1 mice (30–40 g; Charles River, Wilmington, MA) were used for all described experiments. The mice were housed under standard vivarium conditions with free food and water access. Deep anesthesia was induced with sodium barbiturate (40–50 mg/Kg, administered IP). All experiments were performed at Bloodwork's NW, Seattle, WA, with IACUC approval in accordance with NIH guidelines. For reference, RBT‐1 infusion has previously been shown to induce no change in renal function (BUN, creatinine) or renal histology (Johnson et al., 2017; Johnson & Zager, 2018; Zager, 2015, 2022; Zager et al., 2016; Zager & Johnson, 2021).

### 2.4 Mouse plasma SDC‐1 levels following five diverse injury models

To assess whether a broad spectrum of injuries can release SDC‐1 into the circulation, mice were subjected to one of three different AKI models (maleate, glycerol, and cisplatin nephrotoxicity); isoproterenol‐induced cardiomyopathy/acute heart failure with prerenal azotemia (Johnson & Zager, 2021); or acute endotoxemia (see below). Collected plasma samples were assayed for mouse SDC‐1 by ELISA (ab273165; Abcam, Waltham, Boston). Additionally, blood urea nitrogen (BUN) concentrations were measured (DIUR‐500; Bioassay Systems, San Francisco, CA) as an index of renal function.

#### 2.4.1 Glycerol model of AKI

Four mice were lightly anesthetized with isoflurane and the glycerol model of rhabdomyolysis AKI was induced (8 mL/kg 50% glycerol; administered in two equally divided IM injections into the hind limbs) (Zager et al., 2016). Eighteen hours later, the mice were deeply anesthetized, the abdominal cavities were opened, and terminal vena cava blood samples were obtained. Sacrifice was performed by aortic transection. Four saline injected mice served as controls.

#### 2.4.2 Cisplatin (CP) model of AKI

Five mice received an intraperitoneal injection of CP (15 mg/kg; Sigma; P4394; stock solution, 1 mg/mL saline). Three days later, blood samples were obtained from the abdominal vena cava and the mice were then sacrificed, as noted above. Blood samples from four normal mice served as controls.

#### 2.4.3 Maleate

Four mice were injected with Na maleate, a proximal tubule specific nephrotoxin (Sigma, M5757; 600 mg/kg IP in ~500 μL PBS; ref. (Zager et al., 2008)). Approximately 18 h post maleate injection, terminal blood samples were obtained from the abdominal vena cava, followed by sacrifice as noted above. The plasma SDC‐1 values were compared to those observed in four mice which did not receive prior RBT‐1 preconditioning.

#### 2.4.4 Isoproterenol (Iso) model of cardiac injury

Iso administration is widely used as an experimental model of acute heart failure, resulting from extreme tachycardia, hypotension, and myocyte apoptosis and/or necrosis, and prerenal azotemia (Johnson & Zager, 2021). Five mice received a subcutaneous injection of Iso (50 mg/kg dissolved in PBS; from Sigma; I6504 St. Louis, MO). Eighteen hours postinjection, terminal blood samples were obtained, followed by sacrifice. Blood samples from four normal mice served as controls.

#### 2.4.5 E. coli endotoxin (LPS)

Six mice were injected with E. coli endotoxin (2 mg/kg; 0111:B4; L‐2630; Sigma Chemicals, St. Louis, MO; 150 μL via tail vein). Eighteen hours post LPS injection, terminal blood samples were obtained for SDC‐1 assay, followed by sacrifice, as noted above. The results were compared to those observed in four normal controls.

### 2.5 RBT‐1 preconditioning effect on experimental injury‐induced SDC‐1 release

The following experiments were undertaken to determine whether RBT‐1 preconditioning can mitigate SDC‐1 release following two of the above injury models: maleate‐induced AKI or endotoxemia (LPS). Ten mice were injected with RBT‐1 (1 mg FeS + 1 μmole SnPP; ref. (Zager et al., 2016); from Renibus Therapeutics, Southlake, TX). Approximately 18 h later, they received either maleate or endotoxin injection (*n*, 5 for each). Plasma samples were obtained from these mice and mice that had been injected with maleate or endotoxin without prior RBT‐1 preconditioning (*n*, 5 each). The impact of RBT‐1 preconditioning on LPS‐ or maleate‐ induced plasma SDC‐1 increases was assessed.

To determine whether RBT‐1 independently alters plasma SDC‐1 concentrations, the latter were measured in three normal mice and three mice 18 h post RBT‐1 administration in the absence of any additional interventions.

### 2.6 Assessment of whether RBT‐1 preconditioning mitigates aortic injury/stress responses during AKI

The following experiment was designed to seek further evidence that RBT‐1 preconditioning can decrease vascular (aortic) injury/stress responses. Fifteen mice were divided into three groups: (i) control mice; (ii) maleate injected mice; and (iii) maleate injection, administered 18 h after RBT‐1 administration (*n*, 5–6 per group). Approximately 18 h post maleate injection, the abdominal aortas were rapidly excised, iced, RNA was extracted and assayed for tissue stress markers, NGAL, KIM‐1, and IL‐6 mRNAs by RT‐PCR, as previously described (Johnson & Zager, 2018; Zager et al., 2016). The results were factored by simultaneously obtained GAPDH product. Of note, RBT‐1 was first demonstrated to have no independent effect on the assessed mRNA biomarkers (Johnson et al., 2017; Johnson & Zager, 2018; Zager, 2015, 2022; Zager et al., 2016; Zager & Johnson, 2021). The following reagents were used: cDNA preparations: MMLV Reverse Transcriptase Kit – Invitrogen 28,015–013; Oligo(dT) – Invitrogen 58,862; dNTP – Invitrogen 8228G5; RNase – Invitrogen AM2684. Semiquantitative PCR kit and reagents: Taq DNA Polymerase Kit – Invitrogen 10,342,020 and dNTP – Invitrogen R0192. Primer pairs are presented in Table 2.

**TABLE 2:** Mouse primers used for RT‐PCR analyses.

mRNA	Primer sequences	Product size
HO1	5′‐AAC ACA AAG ACC AGA GTC CCT CAC‐3′	288 bp
	5′‐CCAGAG AAG AGA GCC AGG CAA GAT‐3′	
NQO1	5′‐GAG GTA CTC GAA TCT GAC CTC TA‐3′	254 bp
	5′‐ACT CTC TCA AAC CAG CCT TTC‐3′	
KIM‐1	5′‐GAG AGT GAC AGT GGT CTG TAT TG‐3′	231 bp
	5′‐GTG TGT AGA TGT TGG AGG AGT G‐3′	
NGAL	5′‐AAC ATT TGT TCC AAG CTC CAG GGC‐3′	224 bp
	5′‐CAA AGC GGG TGA AAC GTT CCT TCA‐3′	
IL‐6	5′‐TGCCTTCTTGGGACTGATGC‐3′	610 bp
	5′‐CATAACGACCTAGGTTTGCCG‐3′	
GAPDH	5′‐CTG CCA TTT GCA GTG GCA AAG TGG‐3′	437 bp
	5′‐TTG TCA TGG ATG ACC TTG GCC AGG‐3′	

*Note*: Mouse primers used for quantifying mRNA analytes in mouse aortic tissues.

### 2.7 Potential RBT‐1 effects on Nrf2 pathway activation and vascular ferritin expression

#### 2.7.1 Nrf2 assessments

Ten mice received a tail vein injection of RBT‐1, as noted above. Either 4 or 18 h later (*n* = 5 at each time point), mice were anesthetized, the abdominal cavity was opened, and the abdominal vasculature exposed. The perivascular adipose tissue was gently removed, and then the full‐length abdominal aorta was carefully resected, immediately cooled to 4°C, and total RNA was extracted (RNeasy Kit; Qiagen, Germantown, MD). Nrf2 activation was assessed by measuring the mRNAs of two Nrf2 activated genes [heme oxygenase 1(HO‐1); NAD(P)H quinone dehydrogenase 1(NQO1)]. Aortic RNA samples from five normal mice served as controls. Samples were assayed for HO‐1 and NQO1 mRNAs by RT‐PCR, as previously described (Johnson & Zager, 2018; Zager et al., 2016). The values were factored by simultaneously determined GAPDH product. [Note, these (and the above) assessments were performed on aorta, given that it can be obtained free of potentially contaminating organ parenchyma which could obfuscate vascular results].

#### 2.7.2 Vascular heavy chain ferritin assessments

Previous studies have indicated that RBT‐1 upregulates tissue ferritin expression primarily via increased translation, not gene transcription. Hence, to assess whether RBT‐1 increased vascular heavy chain ferritin expression, it was probed in aortic and renal vascular tissues by immunohistochemistry (IHC). Three mice were injected with RBT‐1 and 18 h later, aorta and left kidneys were removed and fixed in 10% formalin. The tissues were then probed for heavy chain ferritin as described at the end of this Section 2.

#### 2.7.3 SDC‐1 expression, as assessed by IHC

The goal of this experiment was to confirm that SDC‐1 shedding into plasma corresponds with loss of SDC‐1 from endothelial cells. To this end, three mice were lightly anesthetized with isoflurane and then subjected to the glycerol AKI model, as noted above. Three sham injected mice served as controls. Approximately 18 h later, the aortas and kidneys were resected and fixed in 10% formalin. The tissues were then probed for SDC‐1 by IHC (see below). Terminal plasma samples were also assayed for SDC‐1, as noted above.

### 2.8 SDC‐1 and heavy chain ferritin: IHC methodologies

IHC studies were performed by the Fred Hutchinson Cancer Center Experimental Histopathology, an NIH supported shared resource (P30 CA015704). Paraffin sections were cut at 4 microns, air dried at room temperature overnight, and baked at 60^o^C for 1 h. Slides were stained on a Leica BOND Rx auto stainer (Leica, Buffalo Grove, IL) using Leica Bond reagents. Epitope retrieval was performed at 100^o^C for 20 min with Epitope Retrieval Solution 1 (Leica AR9640). Endogenous peroxidase was blocked with 3% H_2_O_2_ for 5 min followed by protein blocking with TCT buffer (0.05 M Tris, 0.15 M NaCl, 0.25% Casein, and 0.1% Tween 20), and 0.05% Proclin 300 at pH 7.6 +/− 0.1. Rabbit anti‐heavy chain ferritin, clone EPR18878 (Abcam ab183781) at a dilution of 1:160 was incubated for 1 h and Refine Rabbit polymer horse radish peroxidase was applied for 12 min, followed by Mixed Refine DAB (Leica DS9800) for 10 min and counterstained with Refine Hematoxylin (Leica DS9800) for 4 min after which slides were dehydrated, cleared, and cover‐slipped with permanent mounting media. The same protocol was followed for SDC‐1 immunohistochemistry with the exception that rat anti‐syndecan‐1, clone 281–2 (BD Biosciences 553,715) at a dilution of 1:100 was incubated for 1 h and Refine Rabbit polymer HRP was applied for 12 min, followed by Mixed Refine DAB (Leica DS9800) for 10 min and counterstained with Refine Hematoxylin (Leica DS9800) for 4 min, followed by slide dehydration, cleared, and cover‐slipped with permanent mounting media, as above. Scanning was performed on the Aperio AT turbo scanner.

### 2.9 Statistics

All values are given as means ±1 SD. The clinical SDC‐1 plasma values are presented as ng/mL. For graphic depiction of clinical SDC‐1 values, concentrations were converted to log base 10 to facilitate fit to scale. Baseline versus 2‐day post‐surgery plasma SDC‐1 values within each individual group were compared by paired Student's *t*‐test. The mouse mRNA data were compared by ANOVA with Tukey's HSD, or unpaired Student's *t*‐test. Significance was judged by a *p* value of <0.05.

## 3 RESULTS

### 3.1 Human SDC‐1 plasma concentrations

**FIGURE 1:**
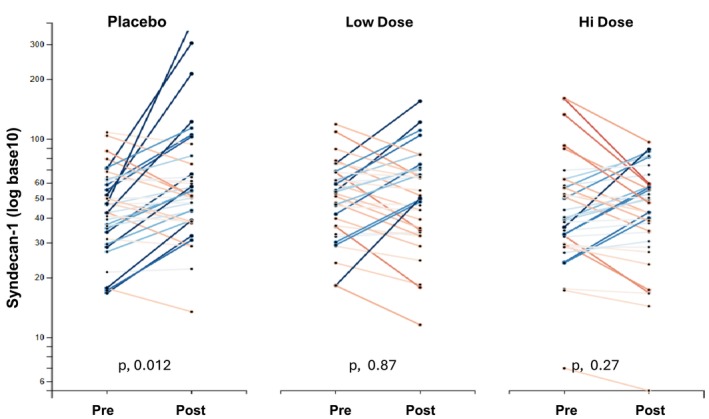
Human plasma SDC‐1 concentrations before and 2 days after cardiac surgery with and without RBT‐1 preconditioning. To fit to scale, plasma SDC‐1 concentrations were converted to log base 10 and pre‐ versus post‐surgery data points for individual subjects in each of the three patient groups are depicted. Pre‐ versus post‐surgery values for each subject are connected by lines. As shown, only the placebo (PBO) group of subjects manifested a significant (*p*, 0.012) postoperative SDC‐1 increase (*p*, 0.012). Non log transformed means and SD values are reported in the Results.

Baseline demographics for the three patient groups are presented in Table 1. Individual subject baseline versus postsurgical plasma SDC‐1 concentrations, converted to log base 10, are illustrated in Figure 1. Mean baseline plasma SDC concentrations (non‐transformed values) did not differ between the three groups (48 ± 18, 53.7 ± 23.4, and 50.5 ± 34.2; ng/mL for PBO, LD, and HD groups, respectively). The PBO group manifested a significant postsurgical plasma SDC‐1 increase over baseline values (from 48 ± 18 to 73.4 ± 66 ng/mL; or 52% increase; *p*, 0.012). In contrast, neither the LD RBT‐1 or the HD RBT‐1 group developed significant postoperative SDC‐1 elevations (LD, from 53.7 ± 23.4 to 57.2 ± 31.2 ng/mL, or 6.4% increase; *p*, 0.47); (HD RBT‐1: from 50.5 ± 34.2 to 56.9 ± 36.0 ng/mL, or 12.7%; *p*, 0.27). Hence, RBT‐1 appears to have mitigated surgery‐induced plasma SDC‐1 increases.

### 3.2 Mouse studies

#### 3.2.1 Plasma SDC‐1 responses to glycerol, cisplatin, and Iso‐induced cardiac stress

Glycerol, cisplatin, and Iso each increased plasma SDC‐1 concentrations over control values (glycerol, 5.1 ± 0.46; cisplatin, 3.8 ± 0.46; Iso, 5.4 ± 2.7; composite controls, 1.6 ± 0.46 ng/mL; each *p* < 0.05 vs. individual sets of controls). Hence, these findings (as well as those from the maleate and endotoxin experiments presented immediately below), confirm that diverse forms of injury in the mouse can evoke marked plasma SDC‐increases. As expected, each injury model caused significant increases in BUN concentrations (glycerol, 89 ± 16; Iso, 86 ± 11; cisplatin, 86 ± 18; composite controls, 22 ± 4 mg/dL; all *p* < 0.05 vs. individual controls).

#### 3.2.2 RBT‐1 preconditioning reduces LPS‐ and maleate‐ mediated SDC‐1 release

Consistent with the above results, LPS and maleate injections also caused significant ~3–4 fold plasma SDC‐1 increases (maleate 4.4 ± 0.4; LPS 5.5 ± 3.0; controls, 1.6 ± 0.4 ng/mL; *p* < 0.05 for each vs. simultaneous controls). The degrees of these increases were significantly blunted by RBT‐1 preconditioning (maleate, 2.8 ± 0.9, *p*, 0.015; LPS, 3.0 ± 0.4, *p*, 0.015 and 0.04 for maleate and LPS without RBT‐1 preconditioning, respectively). Of note, RBT‐1 did not independently alter SDC‐1 concentrations (controls, 1.6 ± 0.4; 18‐h post RBT‐1, 1.5 ± 0.2 ng/mL; NS). RBT‐1 also significantly decreased the degree of LPS‐ and maleate‐ induced BUN elevations (LPS, 72 ± 16 mg/dL vs. LPS + RBT‐1, 46 ± 6, *p*, 0.04; maleate, 131 ± 20 vs. maleate + RBT‐1, 52 ± 22, *p*, 0.01).

#### 3.2.3 RBT‐1 preconditioning protects the aorta from maleate‐induced injury, as assessed by NGAL, KIM‐1, and IL‐6 mRNAs

**FIGURE 2:**
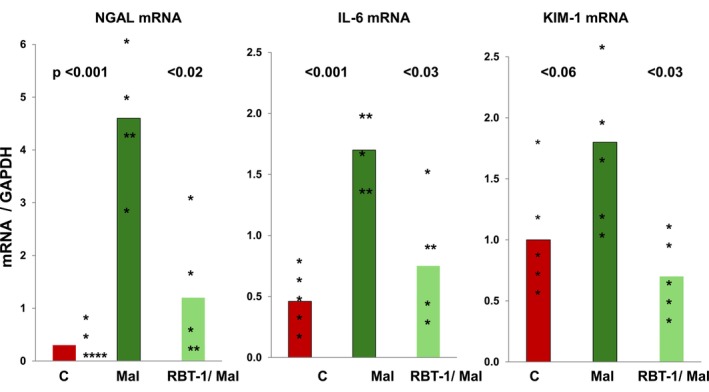
Maleate‐induced injury and inflammatory gene expression in mouse aorta. NGAL, IL‐6, and KIM‐1 mRNAs rose in response to maleate‐induced AKI, indicating vascular stress. RBT‐1 preconditioning significantly attenuated each of these AKI‐induced increases. In data not shown, RBT‐1 in the absence of renal injury, did not significantly affect NGAL, IL‐6, or KIM‐1 mRNA levels. Statistics were performed post log 10 data conversion.

Maleate significantly increased aortic NGAL, KIM‐1, and IL‐6 mRNAs at 18 h post injection, indicating aortic injury/vascular stress (Figure 2). RBT‐1 preconditioning significantly decreased the extent of these increases, indicating a vasculature protective effect.

#### 3.2.4 Aortic Nrf2 and ferritin responses to RBT‐1 preconditioning

##### Nrf2

**FIGURE 3:**
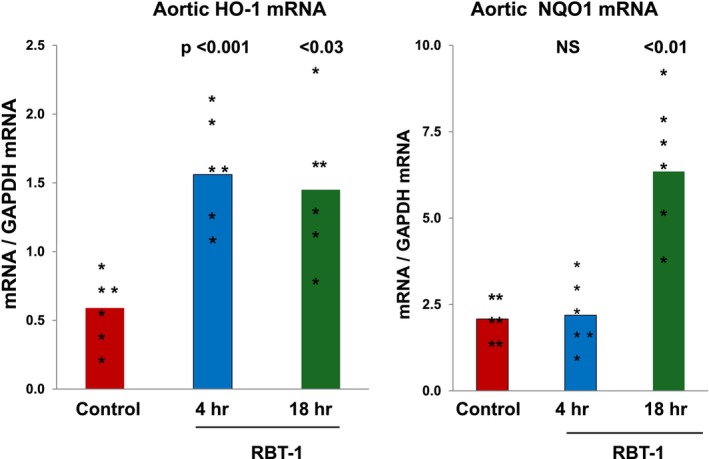
RBT‐1 preconditioning induces Nrf2 activation in mouse abdominal aorta, as assessed by increases in HO‐1 and NQO1 mRNAs. Aortic tissues were extracted for RNA either 4 or 18 h after RBT‐1 or vehicle injection and analyzed for HO‐1 and NQO1 mRNAs. RBT‐1 induced a marked increase in HO‐1 mRNA at 4 h and it remained essentially unchanged at 18 h. RBT‐1 induced a significant increase in NQO1 mRNA at the 18‐h time point.

Within 4 h of RBT‐1 infusion, a 3‐fold HO‐1 mRNA increase was observed and persisted for at least 18 h (*p*, 0.001) (Figure 3). A 3‐fold NQO1 mRNA elevation was evident at the 18 h. (*p*, 0.01), but not at the 4 h, time point (consistent with prior findings that NQO1 reacts more slowly to RBT‐1 vs. other Nrf2 sensitive genes).

##### Ferritin

**FIGURE 4:**
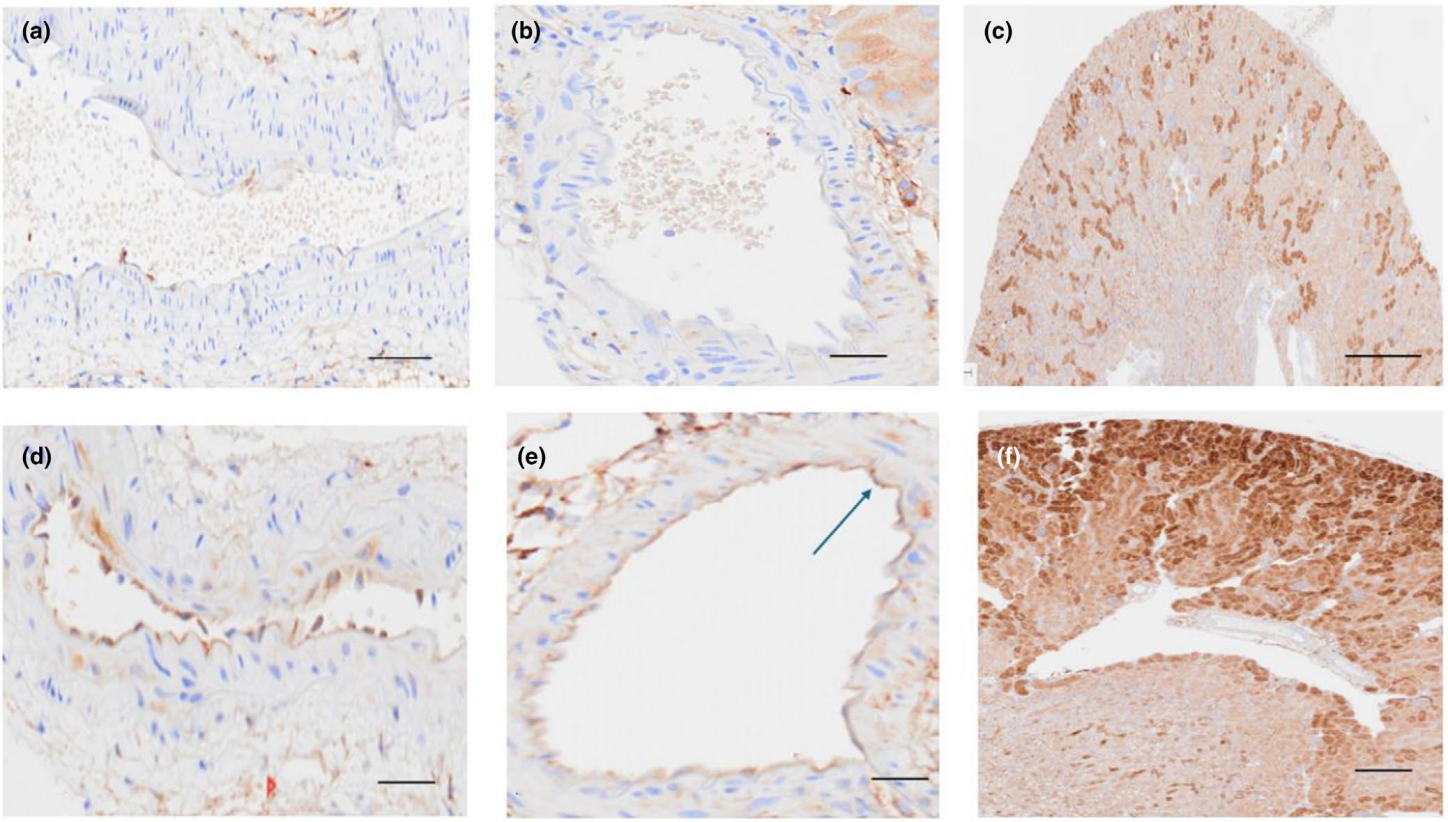
Heavy chain (HC) ferritin expression in aorta, renal segmental artery, and proximal tubules in normal mice and in mice 18 h post RBT‐1 administration. No obvious HC ferritin expression was seen in the normal aorta (panel a) or segmental renal artery (panel b). Conversely, HC ferritin was expressed in both vessels following RBT‐1 injection (aorta panel d; renal segmental artery, panel e; arrow). Whereas foci of proximal tubule ferritin expression were seen in normal kidney (c), it was diffusely and heavily expressed in proximal tubules following RBT‐1 injection (panel f). Scale bars, panels a, b, d, and e, 50 μm; c and f, 200 and 100 μm, respectively.

**FIGURE 5:**
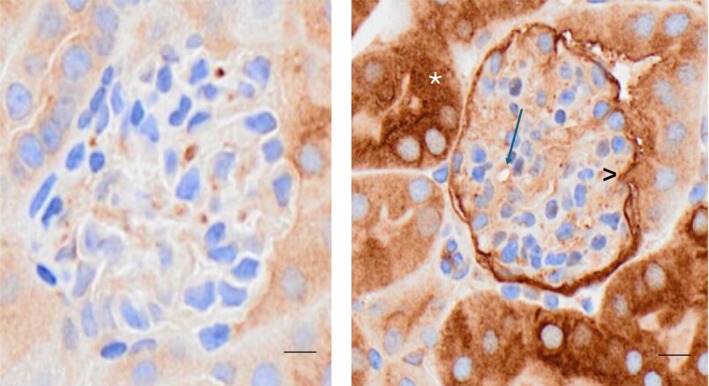
Glomerular ferritin expression in response to RBT‐1 preconditioning. In the absence of RBT‐1 treatment, there were only occasional foci of heavy chain ferritin expression within glomeruli (left hand panel). Conversely, diffuse glomerular ferritin staining was seen throughout glomerular capillaries and the mesangium (right panel). Marked staining of the parietal epithelium (^) and of proximal tubule cells (*) were also observed. Scale bars, 50 μm.

As shown in Figure 4, there was no apparent ferritin staining in normal aortas or in segmental renal arteries (panels a and b, respectively). In contrast, endothelial cell ferritin staining was observed in both vessels following RBT‐1 injection (panels d and e). Low powered view of renal cortices showed marked ferritin increases throughout proximal tubules (panel f). Only occasional ferritin foci were seen in normal renal tissues (panel c). Normal glomeruli showed only occasional ferritin staining. Conversely, RBT‐1‐induced widespread ferritin expression throughout the glomerulus and glomerular capillaries (Figure 5). Unexpectantly, prominent staining of the glomerular parietal epithelium was also observed.

#### 3.2.5 Vascular loss of SDC‐1, as assessed by IHC

**FIGURE 6:**
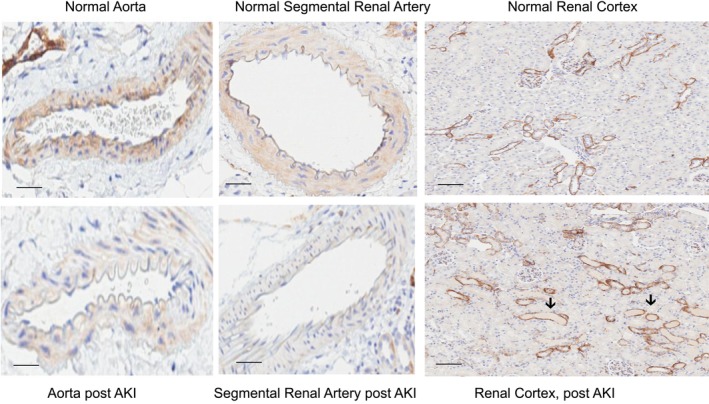
Evidence that AKI can induce SDC‐1 shedding from the vascular endothelium. Both the normal aorta and the normal segmental renal artery demonstrated SDC‐1 staining. Conversely, post induction of glycerol‐induced AKI, there was an apparent absence of aortic and renal segmental artery SDC‐1 staining. In addition, aortic endothelial cell swelling was observed. These observations confirm that AKI can cause vascular SDC‐1 release. No obvious SDC‐1 loss from renal tubules was observed post glycerol AKI, implying that the increase in plasma SDC‐1 levels likely resulted, at least in part, from vascular, rather than renal tubular, SDC‐1 release. The black arrows demonstrate glycerol‐induced heme casts within tubule lumina. Scale bars, vasculature, 50 μm; renal cortex, 100 μm.

Glycerol‐induced AKI caused marked decreases in aortic and renal vascular SDC‐1 staining, compared to normal samples (Figure 6). In contrast, no observable difference in renal tubular SDC‐1 staining was observed between the normal and post glycerol AKI renal tissues. The loss of vascular SDC‐1 post glycerol AKI corresponded with an approximate 4‐fold increase in SDC‐1 plasma concentrations (glycerol, 5.1 ± 0.4 vs. controls, 1.6 ± 0.4; *p* < 0.05).

## 4 DISCUSSION

In a recent phase 2 trial of RBT‐1 preconditioning conducted in patients undergoing “on‐pump” cardiac surgery, several clinical benefits were suggested, including shorter postoperative ventilator times, shorter ICU stays, a decreased incidence of atrial fibrillation, less hypervolemia, less blood product use, and a reduction in 30‐day hospital readmission rates (Lamy et al., 2024). We have previously documented that RBT‐1 activates cytoprotective pathways in multiple organs, including mouse kidney (Johnson et al., 2017; Johnson & Zager, 2018; Zager, 2022; Zager et al., 2016; Zager & Johnson, 2021), liver (Zager, 2015), heart (Zager et al., 2016), and lung (unpublished data). As a result, multiorgan protection against diverse forms of experimental tissue injury has been observed (Johnson et al., 2017; Johnson & Zager, 2018; Zager, 2015, 2022; Zager et al., 2016; Zager & Johnson, 2021). However, the impact of RBT‐1 on the vasculature, and most notably the endothelial glycocalyx, has not previously been explored. It is noteworthy that SDC‐1 and the glycocalyx are critical determinants of vascular function, potentially impacting multi‐organ perfusion, and hence, potential clinical outcomes (Bruegger et al., 2009, 2011; de Melo Bezerra Cavalcante et al., 2016; Dixon et al., 2024; Miyazaki et al., 2024; Pesonen et al., 2019; Salmito et al., 2024; Svennevig et al., 2008; Xu et al., 2021). Thus, we sought to determine whether RBT‐1 preconditioning can reduce SDC‐1 shedding in postoperative cardiac surgery patients, suggesting a vasculature protective effect.

Consistent with the literature (Passov et al., 2021; Pesonen et al., 2019; Svennevig et al., 2008), we observed an approximate 50% increase in plasma SDC‐1 levels at 2 days post cardiac surgery in the placebo group (*p*, 0.012). Conversely, nonsignificant plasma SDC‐1 increases were observed in RBT‐1 preconditioned subjects (placebo vs. RBT‐1 treated subject, *p* < 0.015). To confirm that diverse forms of injury can, indeed, evoke tissue SDC‐1 release into plasma, multiple experimental renal, and extrarenal injury models were studied, and in each case marked plasma SDC‐1 increases were observed. RBT‐1 preconditioning decreased SDC‐1 shedding into plasma by ~50% in each of two experimental injury models (endotoxemia, maleate‐induced AKI), consistent with the above clinical results. Given that SDC‐1 is a widely accepted biomarker of glycocalyx injury, these clinical and experimental data support the concept that RBT‐1 preconditioning can be expressed within vasculature.

To further test whether RBT‐1 might decrease endothelial/vascular injury, NGAL, KIM‐1, and IL‐6 mRNAs were measured in mouse aorta following induction of maleate‐induced AKI. In each instance, RBT‐1 significantly blunted the AKI‐induced mRNA increases. These data are congruent with the RBT‐1 induced reductions in SDC‐1 release, implying a vascular protective effect. Although NGAL and KIM‐1, and their mRNAs, are generally considered markers of tubular cell injury, vascular damage can also increase their expression (Cruz et al., 2012; Sivalingam et al., 2018). This provided the rationale for undertaking the aortic NGAL and KIM‐1 assessments. Of note, we measured NGAL, IL‐6, and KIM‐1 mRNAs, rather than their cognate proteins, given that the mRNAs reflect direct vascular production, whereas their cognate proteins are predominantly generated at extravascular sites, thereby contaminating vascular‐specific targets. Of note, a reviewer of this paper pointed out that although these mRNA increases developed within 18 h of AKI induction, no evidence of aortic leukocyte infiltration or overt inflammation was seen. Nevertheless, aortic SDC shedding and endothelial cell swelling were observed as noted above, consistent with endothelial injury, and hence, the above mRNA results. Whether more overt vascular inflammation, for example, leukocyte infiltration, might have ultimately developed at a later time point remains a possibility.

We have previously documented that RBT‐1 activates the Nrf2 pathway in multiple mouse organs and plays a key role in the RBT‐1 preconditioning response (Johnson et al., 2017; Johnson & Zager, 2018; Zager, 2015, 2022; Zager et al., 2016; Zager & Johnson, 2021). Hence, the question arose as to whether the above noted vascular protection could have resulted from RBT‐1‐induced Nrf2 activation directly within vascular tissues. To address this issue, Nrf2‐ regulated HO‐1 mRNA and NQO1 mRNA levels were measured in mouse aorta, and in both instances, RBT‐1‐mediated HO‐1/NQO1 gene induction was observed. It is notable that Nrf2 activation upregulates a host of antioxidant and anti‐inflammatory genes (Ngo & Duennwald, 2022; Zager et al., 2016). Furthermore, oxidant stress and inflammation are potent inducers of endothelial injury and glycocalyx SDC‐1 shedding (Bruegger et al., 2009, 2011; de Melo Bezerra Cavalcante et al., 2016; Dixon et al., 2024; Gonzalez Rodriguez et al., 2017; Miyazaki et al., 2024; Passov et al., 2021; Pesonen et al., 2019; Puskarich et al., 2016; Salmito et al., 2024; Xu et al., 2021). Thus, it seems plausible that vascular Nrf2 activation could mediate, or contribute to, RBT‐1's vascular protective effect.

Heavy chain ferritin upregulation, which occurs primarily via increased mRNA translation, also contributes to RBT‐1's protective action (Chen et al., 2015). This is primarily driven by RBT‐1's Fe sucrose content. Hence, we assessed whether increased aortic, as well as renal vascular, heavy chain ferritin production might result from RBT‐1 administration. This appears to be the case, given that increased heavy chain ferritin expression was observed in aorta and segmental renal arteries by immunohistochemistry. Widespread ferritin increases were also observed throughout glomeruli, including glomerular capillaries. Given that cellular ferritin exerts a potent antioxidant effect via catalytic iron scavenging, it, along with Nrf2 activation, could confer vasoprotective actions.

It is important to point out several potentially significant limitations of this study. First, although SDC‐1 shedding is widely accepted as a biomarker of endothelial glycocalyx injury, it is notable that SDC‐1 is also expressed in, and can be shed from, epithelial cells, for example, following renal ischemia (Dixon et al., 2024; Guo et al., 2022, 2023; Kajita et al., 2021; Konda et al., 2023). Hence, the relative contributions of endothelial versus epithelial cell SDC‐1 shedding to the observed plasma SDC‐1 increases remain unknown. However, unlike the ischemia AKI model (Guo et al., 2022, 2023), the current glycerol AKI model evoked decreases in aortic and renal segmental artery SDC‐1 staining without any discernable loss of SDC‐1 from renal tubules, as assessed by immunohistochemistry. These findings imply that vascular SDC‐1 release, at a minimum, contributed to the observed plasma SDC‐1 increases. Second, although RBT‐1 activated Nrf2 and upregulated ferritin in mouse aorta, we cannot conclude that these changes occurred throughout the vascular system. Indeed, it is small, rather than large, vessels that control tissue perfusion. Thus, a more thorough assessment of RBT‐1 effects on Nrf2/ferritin expression throughout the vasculature remains an area for future study; Third, although Nrf2 activation and ferritin are known to exert cytoprotective actions on the endothelium (Chen et al., 2015), we do not have direct evidence that Nrf2 and ferritin mechanistically caused or contributed to the RBT‐1‐induced suppression of SDC‐1 release. Additional studies to assess this issue are required. Finally, the ultimate clinical utility of RBT‐1 preconditioning as a method to decrease post‐surgical complications remains to be defined. The results of a recently initiated phase 3, randomized, double blind, placebo controlled international trial of RBT‐1 preconditioning in 450 “on pump” cardiac surgery patients (NCT 06021457) will provide critical information in this regard.

## FUNDING INFORMATION

This work was supported by research funds from Renibus Therapeutics, Southlake, TX.

## CONFLICT OF INTEREST STATEMENT

Both authors are employees of Renibus Therapeutics which provided financial support for the conduct of this work.

## ETHICAL STATEMENT

All procedures that involved human participants were performed in accordance with the ethical standards of the Unites States, Canada, and Australia and in accordance with the 1964 Helsinki Declaration and its amendments and ethical standards. Enrolled subjects provided written informed consent. The examination was made in accordance with all approved principals. All of the preparations and equipment used are officially certified for clinical use.

## Data Availability

Requests for data sharing will need to be cleared by the sponsor, Renibus Therapeutics, but all reasonable requests will be honored.
